# Limitation of life‑sustaining therapies in critically ill patients with COVID‑19: What lessons to draw?

**DOI:** 10.1186/s13054-023-04470-1

**Published:** 2023-05-22

**Authors:** Nicolas Engrand, Armelle Nicolas-Robin

**Affiliations:** Neuro-Intensive Care Unit, Adolphe de Rothschild Foundation Hospital, 29 Rue Manin, 75019 Paris, France

**Keywords:** COVID-19, Pandemic, Limitation of life-sustaining therapies, Ethics, Ethical consulting units

During the first wave of COVID-19, many ethical recommendations were formulated to manage the crisis in the fairest way possible. However, few physicians reported on their experiences in making decisions about the limitation of life-sustaining therapies (LSTs) at the bedside. This is the great merit of the study by Giabicani et al. [1], an ancillary study of the COVID-ICU registry [2], which reports practical facts for 4671 patients from 163 French-speaking centers. This interesting study showed that the main factors leading to LST limitations were advanced age, clinical frailty scale score, and respiratory severity but not intensive care unit (ICU) load. The authors recorded a global period prevalence of in-ICU LST limitation decisions of 14.5%, with a wide variability among centers, from 0 to 30% or even 40% of patients in some centers (see Fig. 3 of the article by Giabicani et al.).

From March 2020 in our neurological institution, we set up an ethical consulting unit and reported our care decision activity for 56 of 111 COVID-19 and 184 neurological patients hospitalized in the ICU and the inpatient department during the first COVID-19 wave [3]. Our experience leads to a few comments.

Giabicani et al. did not report the number of ethical consulting units setup and the overall number of discussions, so the first issues concern the incidence of the option of deciding to not withhold or withdraw treatments. Similarly, the authors did not report the number of advanced decisions of non-admission to the ICU. In our experience, 19% of all our patients benefited from a care plan. Among them, 16% received a decision to pursue intensive care, including for 5% a proposal for re-evaluation of the situation within 48–72 h. We consider that a nonzero incidence of decisions to pursue care would be a guarantee of the quality of collegial deliberations and the primary respect of the patient’s interest in a reasonable therapeutic commitment.

According to our global results, Giabicani et al. reported a fairly high incidence of LST limitations. This result could be due to a low incidence of decisions of non-transfer to an ICU and a deferred LST limitation decision: During this first phase, physicians did not allow themselves to deny access to the ICU for patients with an unknown viral pneumonia because of the hope of saving their lives. This result could be consistent with a “posteriori triage.” However, in the COVID-ICU registry, the number of non-transfer decisions to the ICU was not known, nor were the non-transfer and LST limitation decisions for non-COVID patients. Actually, 44% of patients in our COVID-19 hospital ward and 7.5% in our neurology ward received a decision of no transfer to the ICU. The potentially low rate of non-admission to the ICU of COVID-19 patients in the COVID-ICU registry might explain in part the excess mortality due to myocardial infarction, stroke and cancer, which was + 2.5%, + 8.5% and 1.2 to 1.5 relative risk, respectively, during this period (4, 5) because of the lack of ICU beds for non-COVID patients.

Moreover, this way of deliberating may refer to the tricky issue of decision bias. In the neuro-ICU, where the disability risk is higher than the mortality risk, we are used to deliberating primarily according to functional prognostic goal (modified Rankin score). By contrast, during the first COVID-19 wave, our study showed that according to the COVID-ICU registry, the severity factors (SAPS-2 and specific respiratory or neurological severity) but not frailty factors (age, clinical frailty scale, prior modified Rankin score, Charlson Comorbidity Index) seemed to have influenced our decisions. In the absence of an obvious need to triage patients, we therefore prioritized short-term over long-term survival (i.e., the opportunity to get through the crisis).

Actually, the large heterogeneity in decisions between centers could be related to the field of usual practice of the decision makers, prioritizing the avoidance of disability over the prevention of mortality. Before the SARS-CoV-2 pandemic, such heterogeneity had been outlined [6], depending on ICU end-of-life protocols, palliative care consultations and national end-of-life legislation [7]. Of note, despite a common language and an apparent common medical culture, Switzerland, Belgium and France currently have different approaches and laws concerning the end of life as such (deep and continuous sedation in case of unreasonable obstinacy in France, euthanasia in Belgium and assisted suicide in Switzerland, etc.). These types of decision biases should be studied specifically.

Finally, to explain the decrease in proportion of LST limitations with increasing ICU load, Giabicani et al. propose relevant hypotheses. However, one may also wonder whether this is not just because of the increased bed capacity, which is not taken into account in the “ICU load” concept, and the mathematically decreased need for medical teams to triage.

In conclusion, the Giabicani et al. study shows that the heterogeneity between institutions concerning the organization of the ethical deliberation at the bedside and the resulting LST limitation decisions is striking and undoubtedly challenging. We believe that national recommendations need to be developed in each country, or even international recommendations, to guide and standardize ethical organization in the event of a new pandemic. This development would imply a fundamental reflection, for equitable cure and care between patients with the pandemic disease and other pathologies.

In our opinion, this reflection must also be based on what has been done, in a pragmatic way, that is, by relying on successes and learning from failures, rather than on principles alone, which are undeniably necessary but which can only help decision-making if they are directly applicable “in the field” (e.g., proposing theories that are inapplicable in practice such as prioritarist triage).

These recommendations should probably also promote and guide the development of local ethical consulting units (composition, missions, organization etc.), which would consider the type of local patient recruitment and regional data (urgency of admission, harmonization between neighborhood centers).

## Data Availability

Not applicable.

## Abbreviations

SARS-CoV-2: Severe acute respiratory syndrome coronavirus 2
COVID-19: Coronavirus disease 2019
LST: Life-sustaining therapies
ICU: Intensive Care Unit
